# Transitions in cognitive evolution

**DOI:** 10.1098/rspb.2023.0671

**Published:** 2023-07-12

**Authors:** Andrew B. Barron, Marta Halina, Colin Klein

**Affiliations:** ^1^ School of Natural Sciences, Macquarie University, Sydney, New South Wales, Australia; ^2^ Department of History and Philosophy of Science, University of Cambridge, Cambridge, UK; ^3^ School of Philosophy, The Australian National University, Canberra, Australian Capital Territory, Australia

**Keywords:** unlimited associative learning, comparative cognition, neural networks, major transitions

## Abstract

The evolutionary history of animal cognition appears to involve a few *major transitions:* major changes that opened up new phylogenetic possibilities for cognition. Here, we review and contrast current transitional accounts of cognitive evolution. We discuss how an important feature of an evolutionary transition should be that it changes what is evolvable, so that the possible phenotypic spaces before and after a transition are different. We develop an account of cognitive evolution that focuses on how selection might act on the computational architecture of nervous systems. Selection for operational efficiency or robustness can drive changes in computational architecture that then make new types of cognition evolvable. We propose five major transitions in the evolution of animal nervous systems. Each of these gave rise to a different type of computational architecture that changed the evolvability of a lineage and allowed the evolution of new cognitive capacities. Transitional accounts have value in that they allow a big-picture perspective of macroevolution by focusing on changes that have had major consequences. For cognitive evolution, however, we argue it is most useful to focus on evolutionary changes to the nervous system that changed what is evolvable, rather than to focus on specific cognitive capacities.

## 1. Introduction

The evolution of animals' cognitive capacities, like that of any trait, was a mostly incremental affair [1,2]. Yet some authors have postulated a few larger qualitative jumps—one or more *major transitions* in cognitive evolution. Smith & Szathmáry's [3] work on major transitions in the history of life showed how changes in inheritance opened up phenotypic space in wholly novel ways. Along similar lines, authors have proposed that the development of capacities such as unlimited associative learning [4–6], recursive syntax [7], trial-and-error exploration [8], use of symbols in mental programmes [9] or joint intentionality [10] had a profound effect on the myriad of more specific cognitive capacities that subsequently evolved.

Yet applying the concept of a major transition to cognition faces several serious challenges. The proposals for transitions are, collectively, theoretically heterogeneous. No doubt each postulated capacity is individually important. Yet cognitive capacities are deeply interrelated. Without an agreed-upon cognitive ontology [11], it is difficult to know which cognitive traits are basic and which are derived. The list of proposed transitions also skews towards striking changes, particularly those that make humans unique. The risk of anthropocentrism looms [12]. It is unclear what principled grounds there might be for accepting any particular proposed transition.

In this paper, we approach the idea of a major transition in cognitive evolution with a fresh eye. Returning to the heart of Smith & Szathmáry's [3] original proposal, we propose to focus on the ways in which changes in the *structural organization of cognitive systems* can change the evolvability of new cognitive capacities. Previous proposals have failed to distinguish between mere large changes and major transitions proper because they have not focused on the sense in which proposed changes open up new phylogenetic space. That is in part because explaining proper transitions presents additional challenges. An expanded space of possibility is beneficial for downstream lineages, but evolution is not prospective. Appreciating downstream benefits does not yet show how a transition could have evolved in the first place [13,14].

After reviewing current transitional proposals, we give five examples of structural changes, each of which have had a profound impact on the possible cognitive capacities of descendants. We show how these structural changes could have arisen via ordinary selection processes, without presupposing that subsequent changes in evolvability are the reason for change. Doing so gives a more abstract and theoretically unified account of major transitions in cognition than has been previously proposed.

## 2. What is a major transition in cognitive evolution?

Smith & Szathmáry's [3,15] influential work on evolutionary transitions gave an account of how life evolved that focused on a few changes in inheritance and organization that had radical consequences for the evolvability of organisms. These changes—such as the shift from independent replicators to chromosomes, or from prokaryotes to eukaryotes, or from protists to multicellular organisms—were more than just evolutionarily significant novelties. Smith and Szathmáry emphasized that changes in the *structural bases of inheritance* alter how evolution works, and in particular on what might be evolvable by downstream lineages. The role of constraints in delimiting possible phylogenetic spaces has long been recognized; similarly so with the important role of *changes* in constraints for opening phylogenetic space [16,17]. Crucially, transitions can be driven by relatively mundane selective pressures, such as selection for efficiency and stability. For example, the evolution of chromosomes could have been driven by selection for linkage groups which reduces intragenomic conflict (linked genes replicate simultaneously) and assortment load (complementary linked genes will be found in the same offspring) [18]. Organisms with chromosomes could subsequently establish gene networks and develop in ways that mere replicators could not manage with the outcome being a radical change in what is evolvable.

Smith & Szathmáry's [3] account has not been without critics [19], and it is not clear that their proposed list of transitions contains all or only the best candidates [20]. Nevertheless, the major transitions framework has proven to be a fruitful way to separate the complex history of life into a series of major themes with myriad variations.

The success of transitional thinking about the history of life has led to similar proposals about transitions in the cognitive evolution of animals [5,7,9,21]. Broadly speaking, these proposals tend to postulate the development of a new and valuable cognitive capacity, either domain-general or domain-specific [22], and then elaborate what became possible to creatures with that capacity. These accounts are often explicitly identified as major transitions in evolution by their proponents.

Among the domain-general suggestions include two classic and influential proposals: Dennett's ‘tower of generate and test’ [21] and Ginsburg & Jablonka's [5,23] ‘major neural transitions'. These proposals tend to be hierarchical. Dennett's tower of generate and test, for example, distinguishes four major kinds of creature that differ in their level of cognitive sophistication. Darwinian creatures can only adapt by natural selection; Skinnerian creatures add conditioning; Popperian creatures add internal modelling; finally, Gregorian creatures add language and social modelling. Each level adds a new, broad form of learning and planning that is obviously valuable across a variety of domains.

Ginsburg & Jablonka [5] similarly identify five domain-general transitions: the transition to neurally aided learning in basal animals; elemental associative learning; unlimited associative learning; the development of imagination; and, finally, the use of symbol-based cognition and associative learning. Like Dennett, there are key highlights (the appearance of offline internal models) and a common endpoint (cultural learning). While the transitions are domain-general, they enable a variety of more specific capacities. They argue, for example, that the transition to unlimited associative learning enabled conscious experience [6,23].

Other transitional accounts focus on domain-specific (or at least more restricted) capacities. These tend to non-comprehensive accounts that are particularly focused on human uniqueness and are often only implicitly transitional, as they are not concerned with taxonomizing multiple changes. Hauser *et al*. [7] and Dehaene *et al*. [9] both focus on symbolic language as transformative of human cognition. Dor [24] suggests language and an earlier development of *mimesis* as the two major transitions that enabled human uniqueness. Tomasello [10] suggests joint intentionality as a distinctive enabling feature of the primate lineage. Stout [25] notes a similar role for refined hierarchical action control in the primate lineage. While many of these accounts are human-focused, not all are: Graziano's [26] account of the turning point of attention schemas enabled by the development of the vertebrate pallium arguably counts as well. These accounts are transitional in the sense that they identify large-scale patterns in the history of life.

We agree that all of the innovations cited above are important, and clearly enabled cognitive processes that were not possible before. But should we count them as *major* transitions, or merely important novel adaptations? The dispute is more than a verbal one. For one, there is a real risk of anthropocentrism in highlighting changes that seem important to us: major transitions are not just the ones we find striking. For another, transitional accounts that focus on particular capacities face a difficult dilemma. If the new capacity arose because it was beneficial at the time, this seems like ordinary natural selection: major transitions are not needed. On the other hand, if the new capacity benefitted only downstream lineages by what it enabled, then it remains a mystery as to how or why the capacity evolved. Evolution is not prospective. The problem has been noted to be particularly acute for human-centric transitions [27]: language is great once everyone has it, but why would it arise de novo to enable that future?

We suggest that a more promising alternative is to avoid thinking of transitions as changes in capacities at all. Szathmáry and Smith's account of major transitions in evolution focused on changes in the structural basis of inheritance. Similarly, we propose that major transitions in *cognition* should be identified with structural changes in the systems that implement cognition, which in turn ultimately change how cognitive systems can evolve. Structural changes in neural systems change the computational architectures that neural systems support and enable.

By ‘computational architecture’ we mean something relatively coarse-grained [28] and broadly applicable to the information processing done by nervous systems. A computational architecture in this sense refers to the basic operations, representations, memory and control flow that are available to build computational processes [29]. We focus on animal cognition, and hence the cognitive architecture of nervous systems, but the concept could be applied to other information processing systems too.

We are particularly interested in differences in control flow and memory. As Granger [30] points out, the vast difference in computational power between (e.g.) finite state automata and Turing machines [31] is largely a matter of the difference in how the control flow of programmes can be shaped by the different memory available. Similarly, we suggest that the most important factor for comparing the architectures of different brains is the resources available for shaping control flow. Control flow determines how information moves through brains to be transformed from sensation to motor output, how different parts of the nervous system coordinate with one another [32], and how information can be preserved through various sorts of learning to later shape behaviour. Control flow becomes an increasingly difficult problem as brains get more complex, especially given the reuse of modules for new functions [33,34].

Hence changes in control flow are important for determining what specific cognitive capacities are evolvable. Advances in connectomics allow us to describe brains in terms of the organizational features that determine control flow. Shih *et al.* [35,36] have described the *Drosophila* brain in terms of information flow, which is a key feature of control flow. Their description emphasizes interconnected bidirectional motor and premotor loops that connect the major systems of the *Drosophila* brain [35]. The control flow of the fly brain is recurrent, iterating through motor and premotor loops. Contrast this with similar analyses of the nematode (*Caenorhabditis elegans*) brain where the control flow is strongly centralized and feed-forward [37]. This does not mean there are no recurrent connections in the nematode brain: there are [38,39]. However, the overall control flow of the nematode is feed-forward and could be described as a smooth sensorimotor transformation [37].

Hence structural differences between the brains of *Drosophila* and *C. elegans* correspond to an important difference in the computational architecture of the two cognitive systems. It is these changes we propose to identify as major transitions. Many such structural changes might correspond to major transitions. We flesh out the story by describing five we think are especially important.

## 3. Five types of computational architecture

**Figure 1.**
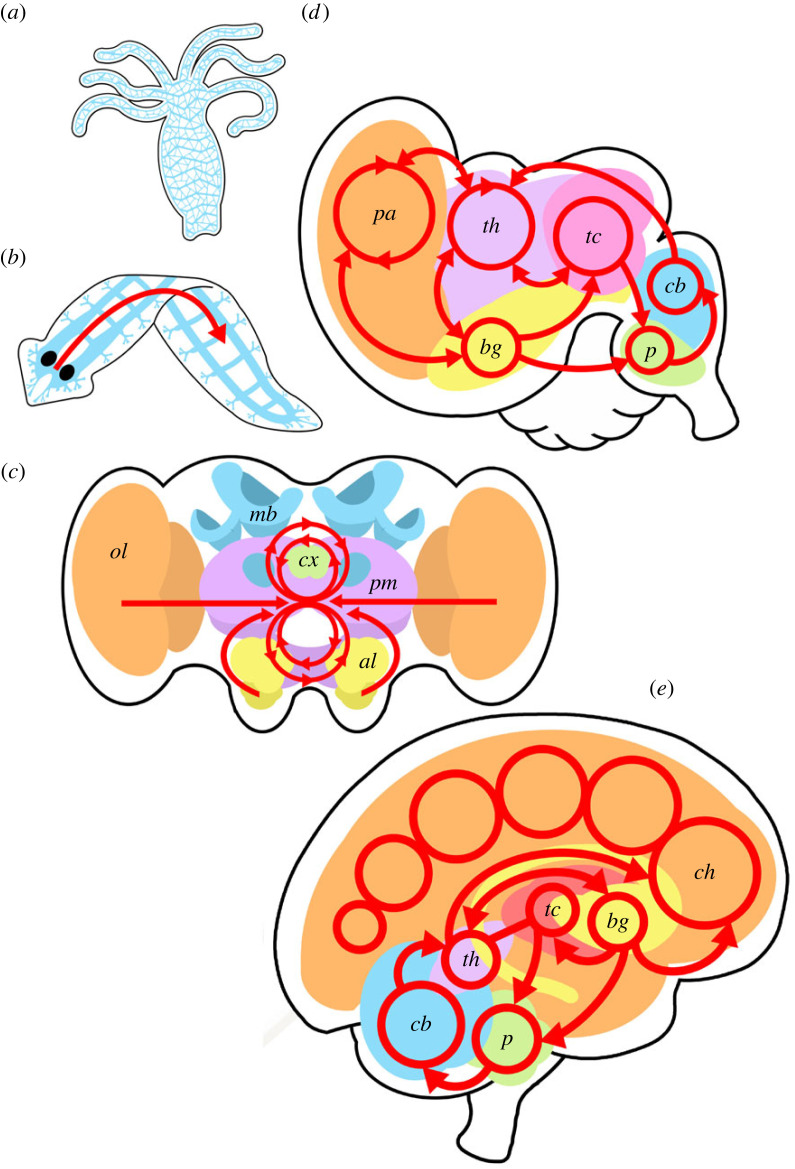
*Five types of computational architecture*. The hydra *Hydra vulgaris* (*a*) has a *decentralized* computational architecture. Its nervous system is a diffuse neural net within which control flow is best described as local and distributed sensorimotor transformation. An example of a *centralized* nervous system is the flatworm *Dugesia japonica* (*b*). The nervous system has bilaterally symmetrical cephalic ganglia which receive input from the major sense organs and coordinate the activity of the body via parallel longitudinal nerve cords. The control flow is dominated by a centralized feed-forward sensorimotor transformation, with the output from the brain delivering commands to coordinate the body (red arrow). (*c*) A simplified frontal section of an insect brain. Within the insect brain are sensory lobes (optic lobe *ol* and antennal lobe *al*), integration centres (mushroom bodies *mb* and central complex *cx*) and premotor centres (*pm*) including the dorsomedial protocerebrum and lateral accessory lobes. The control flow (adapted from [35]) is dominated by bidirectional recurrent loops that connect the sensory and premotor systems (red), hence this is an example of a *recurrent* computational architecture. (*d*) Simplified sagittal section of an avian brain. The control flow involves recurrent connections linking the pallium (*pa*), thalamus (*th*), tectum (*tc*), nuclei of the basal ganglia (*bg*), pontine nuclei (*p*) and cerebellum (*cb*). Each of these regions contains recurrent systems (red). In this *laminated* computational architecture, the control flow of any specific task can be optimized through learning to improve efficiency. (*e*) Simplified sagittal section of a human brain. In addition to the control flow in (*d*), there are virtual control representations generated by computation in structures of the cerebral hemispheres (*ch*) (particularly the frontal and orbitofrontal lobes) that can modify the control flow to improve both the execution of a task and the efficiency of control flow for a specific task. This ability of the human computational architecture to modify its own architecture and control flow is *reflection*.

Emphasizing structural changes, and changes in control flow in particular, gives us a way to compare the computational architecture of brains. With this approach, we suggest that there have been at least five different major transitions, reflected in the presence of five different types of computational architecture across animal brains (figure 1). These we outline below, discussing the new spaces of cognitive possibility that have been made possible by the evolution of each of these types of brain. In the next section, we consider how these computational architectures evolved.

The first transition is from the lack of a nervous system to simple *decentralized* computational architectures. In single celled or syncytial organisms information could move through the body by diffusion or active chemical transport. In these organisms, there are cellular mechanisms for environmental sensing, responsiveness and phenotypic adaptation that have been described as forms of cellular cognition [40,41]. Some unicellular organisms, such as *Stentor coeruleus* demonstrate simple non-associative forms of learning such as habituation using cellular mechanisms of sensing and plastic adaptation [42]. Multicellularity allows for larger and differentiated bodies, which in turn allows organisms to move into different niches and exploit new physical domains and new ways of moving [15]. Multicellularity also presents barriers to chemical communication and to coordination of a larger body, however. Nerve networks are thought to have evolved as a response to selection for faster and more effective coordination of a multicellular body [43]. The Cnidaria (including the jellyfish *Aglantha digitale*, the coral *Acropora millipora* and the hydra *Hydra vulgaris*) is considered a basal animal group [43,44]. The nervous system of Cnidaria has no central brain, and the control flow of the organism is organized by local interactions between sensory elements and local effectors [43,45]. Nerve networks are anatomically diffuse but not random and not without differentiation [46]. Most of the cellular machinery vital to nerve cells, such as voltage- and ligand-gated ion channels, predates the evolution of the nervous system and were part of mechanisms of environmental sensing and homeostatic control in non-neural organisms [47]. Current evidence suggests nervous systems may have evolved at least twice [48]. Decentralized control flow in multicellular organisms can support basic forms of learning: sensitization, habituation and elemental forms of associative learning [49,50]. Currently, sensitization and habituation have been documented in hydra, jellyfish and sea anemones, while forms of associative learning have been reported in sea anemones [49].

In the simple bilaterian phyla such as the Nematoda, Tardigrada and Platyhelminthes we recognize a second transition to a *centralized* computational architecture. Here there is a distinction between a central and peripheral nervous system. The central nervous system contains one or more ganglia linked by nerve cords. The primary ganglion, or brain, is located in the head, with sense organs clustered there for efficiency [47]. The control flow is dominated by a feed-forward sensorimotor transformation [37]. The brain acts as a ‘master controller’ of the body, setting the behavioural priority, coordinating action and physiological states as well as coordinating activities of the limbs [51–55]. A centralized brain thus enables both global coordination and local specialization. Complex, limbed and differentiated bodies are only possible with a centralized computational architecture [43].

Centralized computational architectures equip organisms with a unified decision system that can integrate sensory information via feed-forward neural networks [43]. These can combine inputs across layered neural networks similar to perceptrons [43] or convolutional neural networks. Evidence from worms shows that this more advanced computational architecture allows new forms of learning to evolve, including multimodal learning, cross-modal recall, generalization and classification of information, context learning, latent inhibition, blocking and patterning [50,56–60].

Insect brains have a control flow that is as much feedback as it is feed-forward; hence we recognize a third transition to a *recurrent* computational architecture. Recurrent computational architecture allows the output of a process to be fed back to influence and control the operation of earlier processes. In the insect brain information flow iterates through the modules of the brain in the process of action selection [35,61]. This transforms the space of possible cognitive capacities. In the insect brain representations can reverberate, thereby remaining active and influential in the network over time. Reverberation enables new types of working memory, which in turn supports the learning of relationships between stimuli separated in time [62]. A wider range of relationships can be recognized and learned, including learning of simple ‘abstract’ relationships [63,64]. Coupled fast scan and slower fixation systems can operate on the same information supporting forms of selective attention [65]. Additionally, the output of a sensorimotor transformation can be fed back into the system allowing use of an efference copy to cancel out the consequences of the movement of the sensory systems [66], as well as elementary forms of forward modelling of the consequences of choices and actions [67].

In the vertebrates, and cephalopod gastropods, we recognize a fourth transition to *laminated* computational architecture. In laminated systems, the control flow operates in parallel but interacting recurrent subsystems. Lamination is a structural rather than a physical concept: the avian pallium likely has an equivalent computational architecture to the vertebrate neocortex, but the avian pallium is organized as nuclei while the mammalian neocortex displays layers [68,69]. The important feature is the possibility of multiple parallel but interacting subsystems.

If we trace the control flow in the process of vertebrate action selection we find a process that iterates through interacting parallel systems of the vertebrate brain [1,70,71]. In mammals, the lamination is particularly developed with control flow involving looping interactions between cortical, subcortical, midbrain and cerebellar structures [70]. Each of these major regions contains recurrent subsystems devoted to complex forms of internal and external control [70,72].

Laminated systems can perform multiple operations on the same information at the same time and vary the control flow as a consequence of the outputs of the interacting subsystems. For example, the vertebrate cortex and the cerebellum are connected via multiple subcortical and thalamocortical loops to select an appropriate behavioural priority [73–75] and then fine-tune the exact behavioural response to the situation [70,71,76–78]. The basal ganglia and cerebellum both connect with the cortex through recurrent systems. Basal ganglia–cortex systems identify opportune goals, and cerebellum–cortex systems generate and refine the motor responses necessary to achieve those goals [71]. Operating together these systems promote learning based on practice, and with practice the control flow for motor responses migrates (to a degree) from the cortex to the cerebellum [71,79,80].

Computationally speaking, lamination allows for massively parallel high-dimensional representation of information [81,82], which in turn opens up new classification strategies [76,77]. Lamination allows for representation and control across multiple timescales. It allows information to be integrated and processed in parallel across different spatial and temporal scales. It also allows detailed modelling of the consequences of action across different timescales, enabling forms of prediction and anticipation prior to action. This may apply both to one's own actions and the actions of others [70,71,76,77]. The expansion of the prefrontal cortex in mammals has enhanced the capacity for parallel processing of the same information, expanding capacities for the abstraction of information, operating with sequences of information or the production of complex sequences of behaviour, and forward modelling of the consequences of action [2,70].

Lamination also makes possible distinct use-independent decoupled representations, which in turn facilitates the use of the same representation for distinct purposes (multiplexing) [34]. Note that the advantages of multiplexing are not straightforward. Multiplexing is a very efficient use of neural representations, but it comes at the risk of crosstalk and interference if more than one process is making simultaneous demands on the same representation [34]. Multiplexing then imposes a limit on multitasking [34,83]. Because the control flow in a laminated system has a degree of flexibility and can be modified by learning it can allow systems competing for the same representation to operate through distinct paths of control flow. This provides a partial solution to the problem of crosstalk and interference.

Lamination also increases evolvability. The presence of multiple independent processing pathways allows for a degree of redundancy and degeneracy that allows for new cortical functions to evolve [84,85]. In a laminated system, multiple degenerate pathways are involved in the same process. It is possible for pathways to diverge and adopt different functions, through either neuroplasticity or random evolutionary processes, without compromising the original functions of the system [33]. This facilitates the evolution of novel cognitive functions through the reuse and redeployment of existing systems [33].

The final transition we recognize is a movement to an architecture capable of *reflection*. In computer science, ‘reflection’ refers to the ability of a program to access and modify its own source code [86,87]. This allows a computational architecture to modify its architecture and control flow according to task need. Put another way, a reflective computational architecture can form representations of control flow that affect the control flow itself. Classical stored-program computers have this feature, though self-modifying code is rare. Reflection enables unparalleled flexibility, for it allows the informational architecture of a machine to be specified virtually rather than physically [88].

The benefits of a reflective architecture are increased capacity to adapt to new task demands, combined with increased efficiency in the execution of those tasks. Learning can operate not just on problem solutions, but on the most effective control flow to reach those solutions. These benefits are most apparent for tasks that are complex and hierarchically structured.

Reflection also opens the door to the efficient use of symbolic languages. The development of language was the final proposed major transition in Szathmáry & Smith [15]. We remain neutral on whether the transition to reflection requires natural language or vice versa. However, it is clear that once symbolic languages are in play new forms of reflection then become possible, offering further efficiencies in task acquisition and execution. Language can be seen as a way to compress instructions into simpler forms [89] and enable new types of mental programmes [9]. By a ‘programme’, we mean any way in which control flow can be effectively represented and manipulated, thereby shifting the burden of developing new control flow from the biological to cognitive. Fiebach & Schubotz [90] have proposed that Broca's area (a region of the cortex strongly linked with language and music processing) is ancestrally a region particularly adept at hypersequential processing or recognizing patterns and causality in temporally extended sequences. This is sympathetic with our view of how language has come about. Pain [91] has noted that this type of capacity supports both tool manufacture and language, and could explain why there is such interesting overlap between the brain regions involved in these different kinds of cognition.

Natural language also provides an efficient means for externalizing control flow by distributing complexity among groups of individuals, allowing groups to perform tasks too complex for any individual [92]. Dor [24] has proposed that language enables ‘collaborative computation’ between groups of individuals. This crucially involves treating utterances not merely as informative messages but as *instructions* which can in turn influence control flow. Collaborative work can enable the small-scale apprentice learning that allows for technological transfer and cooperative hunting [93]. On a broader scale, our ability to distribute computational labour allows for complex computations that would be beyond the grasp of any particular human. Reflection thus enables distinctively human forms of learning and cultural accumulation.

We thus recognize five basic important structural changes in animal brains. As with transitions in the history of life [3], we suggest that our transitions are cumulative but not progressive. The power of recurrence, for example, only comes in the context of a centralized feed-forward network. Yet developing recurrence is a change that does not necessarily make organisms better or worse off—a bigger brain is not always better [94]. Simpler systems are more robust; a nematode can survive damage that would cripple anything more complex. Honeybees navigate their environment effectively after just a few short learning flights [95,96], and communicate effectively via their innate dance communication [97]. Prodigious cognitive flexibility is of little benefit to a small short-lived animal, and simpler brains are well-honed to the tasks they need to perform. Our transitional framework is not a stack with humans at the top—it is a tree, with movement towards complexity driven in part by suitability to a niche [98].

## 4. Explaining major transitions in cognitive evolution

Each of our five major transitions changes the computational architecture of cognition, which makes possible new kinds of cognition and learning. A crucial feature of our account is that structural transitions can, at least in principle, be explained in a way that avoids the teleological fallacy [14] of appeal to later benefits to explain earlier changes. Explaining cognitive transitions is necessarily speculative: brains do not fossilize, and gaps in the evolutionary record make it difficult to provide detailed explanations of how large-scale patterns actually arose [99]. We aspire to ‘how possibly’ explanations [100,101], which supply fruitful hypotheses for empirical tests of how major transitions may occur [20,102].

We suggest that resource constraints were drivers of the major transitions proposed above, and that such transitions primarily occur as a way to improve the efficiency of existing functions. As Calcott [103] points out, resource considerations often place constraints on biological design space, making ‘forced solutions’ common (see also [104]). For example, computer models suggest that the early evolution of vascular plants (tracheophytes) involved optimizing across several biological tasks like maintaining mechanical stability, conserving water and intercepting light [105]. Physical and chemical factors play a key role in explaining the evolution of cardiovascular systems in animals [106]. Similarly so with cognition. Brains are energetically expensive and have evolved under extremely tight metabolic constraints. In humans, the brain is 2% of body mass but accounts for 20% of daily energy use [107]. Sterling & Laughlin note that many strategies—like local processing, sparse coding and wire-minimizing topologies—seem to be forced solutions to metabolic demands.

Hence one should expect considerable evolutionary pressure to do the same cognitive tasks more efficiently. From this perspective, the transition is best understood as driven by resource constraints with subsequent changes in architecture opening up phenotypic space. Resource explanations for major transitions are not difficult to generate. For example, all organisms need to coordinate internal processes, and the larger an organism is the harder coordination becomes. A decentralized neural architecture allows different parts of an organism to coordinate quickly and over long distances, thereby overcoming the slow speed and limited specificity of chemical diffusion. Centralization allows for a more efficient network structure once multiple sensory organs are in place with different kinds of information that need to be integrated—a star topology for a network adds new connections more efficiently than does an all-to-all network. Recurrent networks trade space for time, allowing for more compact processing than equivalent ‘unrolled’ networks [108–110]. Recurrence is thus favoured when the energetic costs of recurrence are less than those of adding new layers to a feed-forward network [111].

In each case, we hypothesize the main driver was resource efficiency: after the transition, the lineage may not have gained any new cognitive functions at all. Rather, it was able to do the same thing more efficiently. Yet the effect of that transition on evolvability can be considerable. Decentralized neural networks need to be in place to control large bodies. Indeed, while we often think of nervous systems as fundamentally performing sensorimotor transformations, they play an equally important role in the internal coordination of muscular and glandular activity as the body gets larger [32,112]. Similarly, the development of recurrence opens up a new class of computational functions, as recurrent networks are more powerful than feed-forward networks under biologically realistic conditions [113]. For example, there is a large class of ‘anytime algorithms’ that approximate functions with less error the more they are run [114]. Recurrent networks can flexibly determine how many loops are needed to optimally minimize error given task demands. What the recurrent network can do with time, a feed-forward network must do with space and processors—and using up time is often a more survivable constraint than tolerating the ongoing metabolic costs of more layers.

That said, we emphasize that transitions are not costless. Centralized systems are better at integrating information, but far more vulnerable to catastrophic damage. Decentralized networks are also maximally flexible: they allow any point to be linked to any other, rather than through the common ganglion. Although the recurrent architecture saves on energy, it does this at the expense of other costs: such networks are harder to train, for instance [115]. So it is often a live question as to which strategy is more efficient and effective for a lineage given its actual environment and demands.

## 5. Cognitive transitions and evolvability

We propose that unremarkable selective forces could be drivers of a cognitive transition, but once a transition has occurred it shifts the phenotypic space in at least two ways: first, changing what is computationally possible for an organism and, second, contributing to the evolvability of a population by preserving its standing genetic variation.

According to our account, organisms on either side of a transition boundary might have similar cognitive capacities, but very different potential for evolving *new* capacities. For example, once a population of organisms has a basic decentralized neural architecture, differentiated networks may evolve. Evidence for this is found in the hydra, which has three non-overlapping functional neural networks (identified as groups of neurons that fire within the same 100 ms frame) [46]. Each network is responsible for a different behaviour: radial contraction (which reduces the animal's radius), longitudinal contraction (which contracts the animal into a tight ball) and elongation of the body in response to light [46]. Given design and resource constraints, it is unlikely that an organism relying on chemical diffusion alone would evolve several specialized neural networks without first having a basic network in place that could then be differentiated.

Similarly, a centralized neural architecture facilitates the evolution of capacities like global coordination, multimodal learning and complex sensors. Such features would be too costly or otherwise unlikely to evolve (due to genetic, developmental and other constraints) in a population whose members have only decentralized architectures. Our major transitions are ‘major’ in large part because they make possible numerous new functions and behaviours that were not within reach of organisms with a pre-transition architecture. Phrased in terms of evolvability: the internal features of a post-transition population are such that they are more likely to evolve one suite of cognitive capacities over another (i.e. those found in pre-transition populations) [116–118].

As we argued in §3, each of our proposed transitions has the potential to give rise to new forms of learning. A decentralized architecture supports the evolution of sensitization and habituation; a centralized architecture makes possible the emergence of cross-modal recall and latent inhibition; a recurrent architecture facilitates the evolution of learning sequences and relationships across time. Learning is frequently adaptive, particularly for individuals living in variable environments. Our proposed transitions thus lead to the evolvability of a multitude of adaptive behaviours. These adaptive behaviours do not explain why these transitions occurred, but explain in part how post-transition architectures later become entrenched.

There is an additional important connection between learning and evolvability: learning contributes to evolvability by preserving a population's standing genetic variation [119]. Learning allows a population to adapt to changes in the environment while maintaining genetic diversity. A population unable to adapt to an environmental change through learning must adapt in some other way, such as rapid evolution. In such cases, those individuals who possess the traits needed to survive will do so. This strategy results in a loss of genetic diversity. The remaining population may be poorly equipped to adapt to the next novel environmental threat. By contrast, populations with large amounts of standing genetic variation are more robust and adaptable. Such populations ‘occupy larger areas of genotypic possibility space than less well-endowed populations' [119]. Insofar as major cognitive transitions make possible new forms of learning, they, therefore, change the evolutionary process itself. Post-transition populations can preserve standing genetic variation in ways not available to pre-transition populations.

A feature of our proposed major transitions is that they are contingently irreversible. Smith & Szathmáry [3] held that a major transition is contingently irreversible in part due to a shift in the level of reproduction. Once the ancestor of mitochondria, for example, transitioned from a free-living prokaryote to being part of a eukaryotic cell, most of its genes transferred to the cell nucleus. This change is unlikely to reverse in a way that allows mitochondria to regain reproductive independence. Our proposed transitions do not involve a similar shift in the reproductive unit. However, transitions in computational architecture do dramatically shift the phenotypic space available to a lineage and this in turn leads to contingent irreversibility via path dependence. Once a lineage starts evolving in this new biological space, we might expect the probability of going back to a pre-transition state to be markedly decreased, simply because the newer functions cannot be readily replicated in the older architecture.

In sum, we suggest that major transitions in the history of evolution are best understood in terms of structural changes in information flow that in turn change the effective cognitive architecture of a neural system. These transitions cannot be identified with any individual cognitive change, any more than the shift to multicellularity can be identified with a particular organism. But such structural changes profoundly change phylogenetic space, which is what marks them as truly major transitions.

## 6. Conclusion

Major transitions do not need to be sudden, or inevitable, or dramatic. Nor do transitions necessarily immediately change what an organism can do. Rather they change the evolvability of descendant lineages. We have argued that the most useful way to consider changes in cognitive evolution is to consider the action of selection on the computational architectures that support cognition. Subsequent evolutionary divergence explores the possibilities that are opened by each major transition.

Unexceptional selective forces, such as selection for efficiency, stability or robustness, could drive the evolution of structural changes in computational architecture, which can change the evolvability of a lineage. Thinking of evolution in terms of major transitions thus provides a larger perspective which emphasizes changes in evolvability enabled by each transition rather than changes in phenotypes at points in time. In our article, we have focused on animal nervous systems, and we have proposed five major transitions in the diversity in animal cognition.

Our transitions are cumulative but not progressive. One is dependent on what came before, but one is not better than what came before. Brains are part of embodied systems that are adapted to different niches. A multicellular organism has lost certain possibilities that remain open for unicellular creatures. Just as the move from unicellular to multicellular life requires individual cells to work together rather than optimize their individual fates, new forms of brain organization bring new demands and new limits as well as new opportunities. Diffuse neural networks are intrinsically parallel, for example, whereas centralization of a network produces a serial bottleneck that later transitions only incompletely overcome. Centralized architectures can work well out of the box, while recurrent architectures require more training to be useful. And, of course, a great number of organisms manage perfectly fine without any brain or nervous system at all. The benefits of transitions are that they help us understand the big picture of cognitive evolution, and they help to structure the diversity of animal minds.

Section one canvassed other proposals for major transitions in cognition. One might reasonably wonder how those accounts map onto ours. The answer is that there is no easy mapping because our transitions are different in kind, not just placement. The debate over cognitive transitions is not merely one about which transitions occurred, but about what a transition amounts to in the first place. We have argued here that changes in computational architecture are compelling candidates for major transitions in cognitive evolution. Our account avoids the teleological fallacy of explaining major transitions in terms of post-transition selective advantages. It shows how commonplace resource constraints can change computational architectures in ways that radically transform subsequent evolution.

A focus on computational architecture has the necessary generality for capturing large-scale patterns in the history of life. Previous accounts have focused on particular cognitive capacities (such as the addition of *mimesis*, language or theory of mind) or methods of adaptation and learning (like Dennett's different styles of problem solving). Yet these capacities depend on a broader computational architecture. For example, kinds of learning concern how the space of possible algorithms can be explored, while computational architecture determines the space itself. Our five major transitions provide a background against which these and other crucial shifts can be taxonomized and contextualized, without being exhausted by any of them.

## Data Availability

This article has no additional data.
